# Dimers of G-Protein Coupled Receptors as Versatile Storage and Response Units

**DOI:** 10.3390/ijms15034856

**Published:** 2014-03-19

**Authors:** Michael S. Parker, Renu Sah, Ambikaipakan Balasubramaniam, Edwards A. Park, Floyd R. Sallee, Steven L. Parker

**Affiliations:** 1 Department of Microbiology and Molecular Cell Sciences, University of Memphis, Memphis, TN 38152, USA; E-Mail: michaelsparker@msn.com; 2 Department of Psychiatry, University of Cincinnati School of Medicine, Cincinnati, OH 45267, USA; E-Mails: sahr@ucmail.uc.edu (R.S.); saleefr@ucmail.uc.edu (F.R.S.); 3 Department of Surgery, University of Cincinnati School of Medicine, Cincinnati, OH 45267, USA; E-Mail: balasua@ucmail.uc.edu; 4 Department of Pharmacology, University of Tennessee Health Science Center, Memphis, TN 38163, USA; E-Mail: epark@uthsc.edu

**Keywords:** heteropentamer, G-protein heterotrimer, heterodimer, homodimer

## Abstract

The status and use of transmembrane, extracellular and intracellular domains in oligomerization of heptahelical G-protein coupled receptors (GPCRs) are reviewed and for transmembrane assemblies also supplemented by new experimental evidence. The transmembrane-linked GPCR oligomers typically have as the minimal unit an asymmetric ~180 kDa pentamer consisting of receptor homodimer or heterodimer and a G-protein αβγ subunit heterotrimer. With neuropeptide Y (NPY) receptors, this assembly is converted to ~90 kDa receptor monomer-Gα complex by receptor and Gα agonists, and dimers/heteropentamers are depleted by neutralization of Gαi subunits by pertussis toxin. Employing gradient centrifugation, quantification and other characterization of GPCR dimers at the level of physically isolated and identified heteropentamers is feasible with labeled agonists that do not dissociate upon solubilization. This is demonstrated with three neuropeptide Y (NPY) receptors and could apply to many receptors that use large peptidic agonists.

## 1. Introduction

Protein oligomerization, a very frequent association of identical or non-identical protein chains, could be viewed as a general paradigm of protein organization. Hetero-oligomers constituted of non-identical functionally cooperating polypeptides (as found in a large number of enzymes), or structurally complementing carrier chain complexes (e.g., hemoglobins) frequently have a functionally indispensable stability, and are not thought of as oligomeric assemblies. A large number of proteins however do not depend upon oligomerization for their principal function, but do oligomerize with (identical, similar or dissimilar) polypeptide counterparts, usually with changes in functional efficacy (in receptor proteins especially a larger affinity for agonists). This includes multitudes of molecules that have extensive hydrophobic segments, including one of the largest categories of sequence in metazoan genomes, the heptahelical G-protein coupled receptors (GPCRs).

An intramembrane *intramolecular* helical bundle is found for bovine rhodopsin [1], a multicellular-eukaryote GPCR. The transmembrane bundling in eukaryote opsins and related non-visual GPCRs depends primarily on hydrophobic and hydrogen bonding, since ionic sidechains are not frequent in their transmembrane (tm) helices. Thus, in 211 human family A (rhodopsin family [2]) GPCRs acidic residues average 2.5%, and basic 4.8% of the total tm amino acid residues, while the respective levels for the rest of the molecule are 8.6% and 16.6%.

An intramembrane *intermolecular* association in GPCRs is also ubiquitous, with specificity that sometimes depends on aromatic sidechains [3,4]. This association is typically supported by hydrophobic and hydrogen bonding (which is weakly electrostatic), and requires a close positioning of protomers. Without participation of stabilizing partners, the resulting complexes are short-lived [5]. However, dimers of heptahelical receptors are principally detected in much more stable complexes with G-proteins (see [6]), the abundant mainly cytosolic transducers. Other non-effector or quasi-effector partners of the receptor dimers can include protein phosphatases [7,8], ion transporters and exchangers ([9]; see also [10]), dynamins [11], receptor activity modifiers (RAMPs; [12,13]), and even ubiquitinated ER proteins [14].

**Figure 1.**
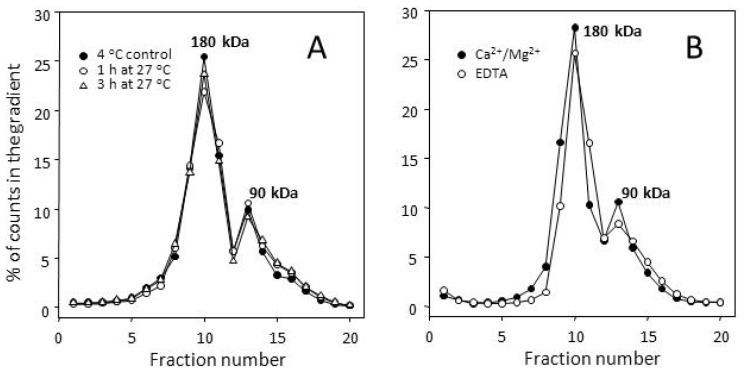
Stability of agonist-labeled Y2 receptor dimers to incubation at 27 °C and to removal of divalent cations. Duplicate 5%–20% gradients (see Section 3.3) were centrifuged for 18 h at 5 °C. All results are average percentages of total counts in the corresponding gradient fractions from two gradients for each condition. The respective standard errors were in most cases below 10%, and for clarity are not shown. (**A**) Y2 receptor dimers in particulates from CHO cells are stable to incubation at 27 °C. The incubation of particulate suspension in the assay buffer at 27 °C was for 1 h (followed by 2 h at 0–4 °C) or 3 h, while the control suspension was incubated in ice for 3 h. This was followed by labeling for 20 min at 27 °C by [^125^*I*]PYY(3–36). The content of dimer, in % total gradient counts, was 66.2 ± 3.7 for 4 °C control, 63.4 ± 3.4 after preincubation for 1 h at 27 °C, and 64.3 ± 3.4 after preincubation for 3 h at 27 °C; (**B**) Y2 receptor dimers are not disbanded by removal of divalent cations. The profiles are for receptors labeled by [^125^*I*]PYY(3–36) (30 min at 25 °C), solubilized and sedimented in buffer A or buffer B. The dimer content, in % gradient counts, was 65.9 ± 4.7 with buffer A, and 60.8 ± 4.6 with buffer B.

The most studied and probably the most frequent complexes of eukaryotic heptahelical receptors are those with G-protein αβγ heterotrimers. With many GPCR dimers these assemblies survive solubilization by steroid detergents such as digitonin and cholate and could be detected as ~180 kDa heteropentamers by density gradient centrifugation ([15,16]; see also Figure 1 in this study) and also can be solubilized by acylamine oxide surfactants such as lauryldimethylamine oxide (LDAO) and detected by circular dichroism spectroscopy [17]. The frequently used zwitterionic detergents, e.g., CHAPS (3-[3-cholamidopropyldimethylammonio]-1-propanesulfonate) tend to destabilize at least the neuropeptide Y (NPY) receptor dimers [18]. Detection of dimers *in situ* is done by fluorescence resonance energy transfer (FRET) (e.g., [19]) and bioluminescence resonance energy transfer (BRET) (e.g., [20]). Variants of the FRET and BRET procedures are by far the most employed in detection of GPCR dimers.

There is an overwhelming experimental evidence for Gαβγ complexes of the peptidic-agonist (and especially the neuropeptide) GPCR dimers as the physiological non-activated state of the receptors, and this also applies to the prototypic rhodopsin (gray opsin) non-peptidic GPCR [21–23]. This type of organization is compatible with an episodic secretion of agonist peptides, and may devolve to Gα-associated monomers in conditions of high G-protein concentration (ref. [24] and this work). Many, if not most, GPCRs responding to monoamines (and especially to catecholamines) are usually detected as monomers associated with Gα subunit, which is the minimal functional unit of GPCR activity [25]. However, this may also relate to the low stability of agonist binding, to high basal levels of circulating aminergic agonists, and to abundance of G-proteins (ref. [26]; also see Figure 4 in this work).

The virtues of oligomerization for GPCRs are many. Oligomers would have much larger ability than monomers to anchor to the membranes, rafts and vesicles. Protein (including the receptor) removal by particulate (proteasomal and/or lysosomal [26]) proteolysis is found to be much less for dimers compared to monomers [6], as is also observed for enzyme carriers [27]. This is dramatically confirmed by elimination of NPY receptor dimers by pertussis toxin via ADP-ribosylation of Gαi subunits [28–30]. Association with transducers and effectors could also be enhanced for the dimers. Chances of successful collision with agonists should increase due to reduced lateral motion and wobbling of dimers compared to monomers. With peptide receptors, affinity of specific agonist peptides for the dimers could be much larger than for the monomers, as indicated by differences in cation sensitivity and cholate extraction (Figure 6 in this work and ref. [15]). In addition to improving the signaling, the high affinity binding to dimers could also help clearance of large agonists. Several lines of evidence converge to indicate that GPCR oligomers serve both as storage [16,31,32] and signal initiation assemblies [23].

In systems rich in Gαβγ heterotrimers containing Gi, Go or Gt α subunits (including visual rods and brain neurons), active GPCRs are solubilized chiefly as Gα-linked monomers (see [23] for rhodopsin, and [16,24] for brain Y1 and Y2 receptors). The ultimate acting unit of GPCRs in many cases could be the monomer [33–35]. However, the much higher affinity for agonists would greatly increase versatility of heteropentamers as the immediate response units. The agonist-activated protomers or monomers could transduce through a monomeric or heteromeric GTPase, or via a non-GTPase molecule or complex, such as the heterodimeric βγ complex [36].

## 2. Results and Discussion

### 2.1. On the Chances of GPCR Homo- and Heterodimerization

Barring a few notable exemptions (such as visual opsins, pineal adrenergic receptors, and kidney epithelial Y2 receptors), GPCRs are characterized by low densities of expression [37]. The endocrine nature of agonist production, release and distribution in metazoa [38–41] implies episodic high-intensity discharges coupled to depletion of surface receptors and to a temporally matching activation of the corresponding genes in proportion to the agonist stimuli. The most likely partner for dimerization during co-translational and post-translational processing in ER/Golgi could be another copy of the same receptor. This may reduce heterodimerization at the level of receptor biosynthesis. It is also reasonable to speculate that most of dimerizing GPCRs did evolve motifs and tracts that support homodimerization. Reassembly of dimers from internalized monomers in endosomes may or may not have similar partnering opportunities. A constitutive, non-transductional dissociation of dimers and re-association of monomers within plasma membrane could also occur. However, this does not seem highly probable for the receptor dimer coupled to G-protein heterotrimer, which would be stabilized by the G-protein subunits, as found for the Y receptors (Figures 1 and 3A).

G-protein-free receptor dimers could associate and dissociate in the bilayer at high rates in an enthalpy-driven way [5,42,43], and could be the main source of heteromers, depending on a synchronic availability of monomers of other receptor species. A majority of such dimers will be of low stability and could have a lower affinity for agonists compared to G-protein coupled dimers. The heterodimerization would also be increased by mass action via receptor overexpression. However, native heterodimers formed under physiological conditions have been demonstrated in a number of studies, and may constitute a significant fraction of oligomeric GPCRs. These hybrids could be of interest in a host of physiological and pathological conditions, as well as in terms of physiochemical properties. Stability and high agonist affinity of Y receptor homodimers [18] suggests comparisons with heterodimers composed of different Y receptors as well as those combining Y and non-Y GPCRs.

### 2.2. Some Notes Related to Methods and Paradigms

Analysis of receptor levels and interactions in the past 15 years was dominated by use of clonal fluorescent or luminescent protist protein attachments. The covalent linking of these attachments ensures that the signal, at least as generated at the plasma membrane, can be considered to properly report on the coupled receptor. This avoids uncertainties linked to steady-state quantitation by attachment e.g., of radioactive blockers to receptors that handle small agonists. In many cases the detection could be done by “flagging” with convenient covalent small peptidic tags (a procedure that preceded the use of fluorescent proteins as markers). However, characterization of these complexes requires solubilization and other handling, such as immunoadsorption and/or immunoblotting, while the receptors labeled by fluorescent/luminescent protein tags in most cases can be examined directly on the cells being studied, which greatly simplifies the assays and reduces the work load. On the other hand, since the size of the attachments has to be large enough to permit an excitation that is sufficient for detection, the proteinaceous tags tend to significantly modify physiochemical properties of the receptors. For instance, the green fluorescent protein from *Aequorea victoria* (GFP; P42212) has two acidic clusters, and *Renilla reniformis* luciferase (Rluc; P27552) has three, and these might influence Gα contact with GPCRs, lowering the agonist affinity [20]. Another problem related to the strictly *in situ* detection of the tags is that nothing is directly known about receptor partners, such as G-proteins and effector cyclases or phospholipases, and behavior of receptor partners is assumed to follow a few consensus patterns, which in some cases may not apply.

Demonstrations of either homo- or heterodimerization frequently use receptor expressions at quite large, even multi-picomolar, levels. From mass law, the larger the inputs of different receptor plasmids, the higher could be the yield of heterodimers. Most of the demonstrations also depend on fluorescent/luminescent signals that only allow detection at the level of fixed cells, and stability of the oligomers, level of oligomerization and association with transducers and effectors are judged by inference or reference. However, in non-induced cellular conditions most GPCRs are expressed at relatively low levels [37], and in physiological tissue settings many native GPCR expressions are at or below 100 fmol/mg particulate protein. From the above considerations, one may object to conditions of the clonal receptor expression and assay that can favor predicted outcomes. Also, it would be instructive to examine receptor homo- and heterodimerization in tissues that have naturally high GPCR levels, such as the pineal gland (for aminergic receptors) and the kidney proximal tubule cells (for the Y2 [44] *vs*. vasopressin receptors).

The native GPCR oligomer levels appear to significantly relate to G-protein levels. The tri-dimensional lattice of rhodopsin multimers in the visual rod cells is established in an environment that has α-transducin at similar levels of mRNA expression [45], and both proteins represent a significant fraction of total cell protein. At the other extreme, rabbit forebrain Y2 NPY receptors in most areas are measured below100 fmol/mg synaptosome protein, while the *G*i/o *B*_max_ values are in the range of 100–200 pmol/mg [24], and most Y2 receptors are detected as monomers (see also Figure 3). In rabbit kidney cortex, the Y2 receptor is natively expressed at about 300 fmol/mg postmitochondrial particulate protein, the *G*i/o *B*_max_ is ~5 pmol/mg, and most Y2 receptors are found as dimers in heteropentamers [24]. The physiologic oligomeric status of GPCRs appears to strongly reflect the transductional environment.

### 2.3. Many Peptide Receptor Dimers Can Be Studied Using the Binding of Native Agonists

The classical view of agonist binding assumes an attachment that is readily terminated by dilution or competition by ligands of higher affinity, such as competitive antagonists. The weak agonist binding for small ligand receptors, such as catecholamine receptors, greatly diminishes analytical options. This has signally contributed to the analytical success of covalent proteinaceous tags. However, many GPCRs with peptidic agonists show agonist attachments that are stable at least at low temperatures, and slow to dissociate even at 37 °C. Such stable (“quasi-irreversible”) binding was found for endothelin at endothelin-1 receptor [46], salmon calcitonin at the human calcitonin receptor [47], pancreatic polypeptide at the Y4 receptor [48], neuromedin U at its receptors [49], NPY at the Y2 receptor [50], PYY(3–36) also at the Y2 receptor [16], and NPY (but not PYY) at the Y1 receptor [51]. At 4 °C, this list ought to expand considerably. This however is much dependent on concentration of the cognate Gα subunits. Evidence was presented previously [24] that the monomerization of forebrain Y receptors occurs largely in the course of the assay. Suppressing Gi activation by complexing of divalent cations could be helpful in extending the applicability of native ligands as markers at least for receptors that do not depend on divalent cations in agonist attachment.

**Figure 2.**
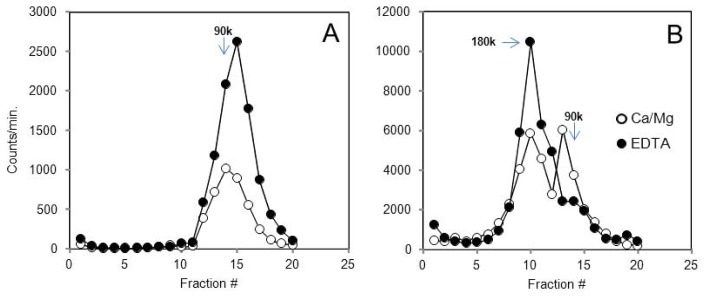
Rundown of the Y2 agonist is much faster in tissues with large *G*i/o content. The labeling at 50 pM [^125^*I*]PYY(3–36) was for 60 min at 25 °C, to allow a slow conversion of dimers to activated Gα-associated monomers and discharge of agonist peptide. The rabbit kidney cortex has a much lower expression of *G*i/o subunits than the anterior hypothalamus (The *B*_max_ for [^35^*S*]GγS binding is more than 20-fold higher in hypothalamic tissue (ref. [24], Table 2). However, the kidney Y2 receptor is expressed at about four times the level of the hypothalamic. The above parameters in the Ca^2+^/Mg^2+^ buffer lead to a much faster transduction with hypothalamic particulates (graph **A**); and a considerable (58%) rundown of the tracer peptide relative to 1 mM EDTA buffer. In the same assay, the relative rundown is only 12% with kidney particulates (graph **B**).

The Y receptor-linked activation of Giα subunits (the principal clients of these receptors) is of course much larger in Gi-rich tissues such as forebrain, and the resulting use of heteropentamers is much faster in particulates from e.g., hypothalamus (Figure 2A) than in those from kidney cortex (Figure 2B). (The *B*_max_ for [^35^S]GγS binding is more than 20-fold higher in hypothalamic tissue [24]. However, the kidney Y2 receptor is expressed at about four times the level of the hypothalamic. The above parameters in the Ca^2+^/Mg^2+^ -containing buffer A lead to a much faster transduction with hypothalamic particulates (Figure 2A), and a considerable (58%) rundown of the tracer peptide relative to 1 mM EDTA -containing buffer B. In the same assay, the rundown is only 12% with kidney particulates (Figure 2B).

**Figure 3.**
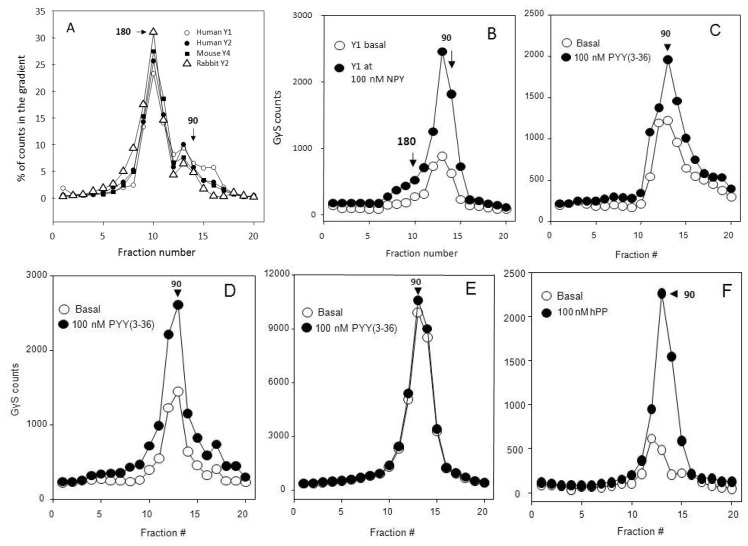
The Y receptors expressed in CHO cells and rabbit kidney Y2 receptors are largely detected as 180 kDa heteropentamers. The rabbit brain Y2 receptors are however in 90 kDa complexes with *G*i/o (see Figure 3 and graph 3E in this figure). The Gi α subunits in heteropentamers (**A**) are activated by respective agonists into 90 kDa complexes with receptor monomers: (**B**) CHO-Y1; (**C**) rabbit kidney Y2; (**D**) CHO-Y2; (**E**) rabbit piriform cortex; (**F**) CHO-Y4. All assays used 50 μg particulate protein.

The heteropentamer/Y2 receptor dimer -attached agonists can be converted to Gi3α-attached Y2 monomers which are immunoprecipitated with antibodies to this subunit, as are the [^35^*S*]guanosine 5′-(γ-thio)triphosphate (GγS) -labeled α subunits associated with the monomer [16]. The G-protein trimer in 180 kDa heteropentamer is not labeled by GγS and is not immunoprecipitated with Gi3α antibody, but does convert to immunoprecipitable 90 kDa Gi3α-receptor monomer at 1–5 mM Mg^2+^ in the presence of micromolar GDP or GγS (Figure 3).

The degree of Gi activation above the basal by a particular receptor agonist is much dependent on densities in the particulates assayed of the cognate receptor and other GPCRs present, and especially the density of the Gα subunits. Thus, as seen in Figure 3, the stimulation above the basal is much less for kidney cortex (graph C), a tissue rich in many types of A-GPCRs, than for CHO cell Y2 expression (graph D), and the activation by rabbit piriform cortexY2 receptors is overwhelmed by contributions of other receptors that produces a very large basal 90 kDa binding of [^35^*S*]GγS (graph E). CHO cell expressions of the human Y1 (graph B) and especially of the human Y4 receptor (graph F) appear to have more extensive activation than CHO-Y2 expression (graph D), which could relate to the large acidic clusters of the Y2 receptor (see [52]).

**Figure 4.**
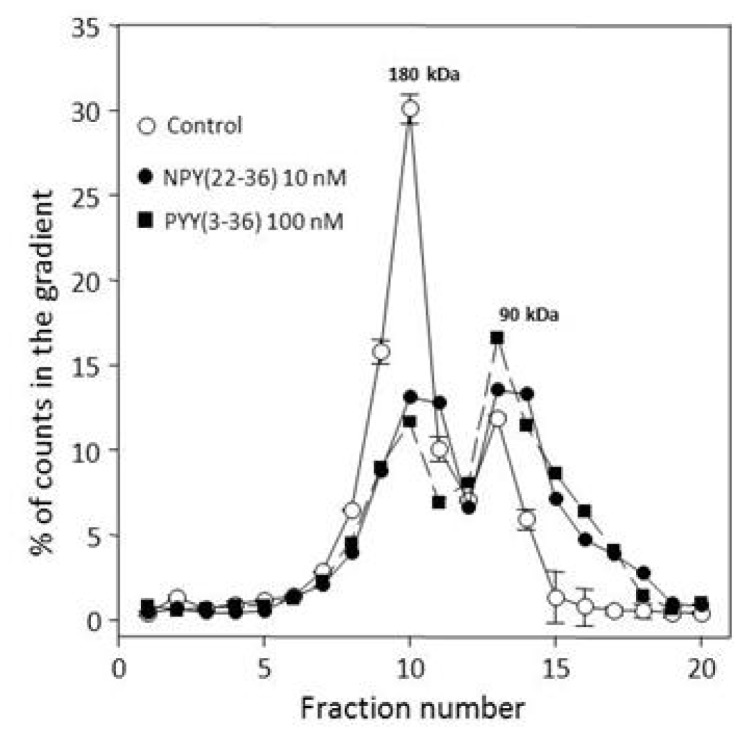
Reduction of Y2 receptors dimers by two agonists of the Y2 receptor. **N**PY(22–36) (K_diss_ *vs*. [^125^*I*]PYY(3–36) at hY2 receptor 6.2 ± 1.2 nM) was used at 10 nM, and PYY(3–36) (K_diss_ at hY2 receptor 0.50 ± 0.06 nM) at 100 nM final, for 45 min at 25 °C prior to resedimentation and labeling with 50 pM [^125^*I*]PYY(3–36). After lysis at 10 mM each of digitonin and cholate, duplicate 5%–20% gradients for each condition were centrifuged for 18 h at 5 °C. All results are average percentages of total counts in the corresponding gradient fractions. The standard errors were in most cases below 10%, and for clarity are shown only for the control profile. The content of dimer, in % total gradient counts, was 67.7 ± 4.8 for control gradients, 42.7 ± 2.3 after pre-treatment with 10 nM NPY(22–36), and 30.5 ± 2 after pre-treatment with 100 nM PYY(3–36).

A similar reduction of CHO-Y2 dimers by pretreatment with two highly specific agonists differing in affinity by more than an order of magnitude and used at very different levels of saturation (Figure 4) shows a large sensitivity of monomerization to agonist levels. This could be of interest in many physiological conditions. The loss of CHO-Y1 and CHO-Y2 dimers due to neutralization of Gi type α subunits through ADP-ribosylation by pertussis toxin [16,29] could be viewed as a constitutive equivalent of the reduction shown in Figure 4. In conditions of normal Gi utilization in CHO-Y2, the dimer reserve is reduced by less than 50% at 100 nM extracellular PYY(336) over 60 min of incubation at 37 °C in the growth medium (data not shown).

**Figure 5.**
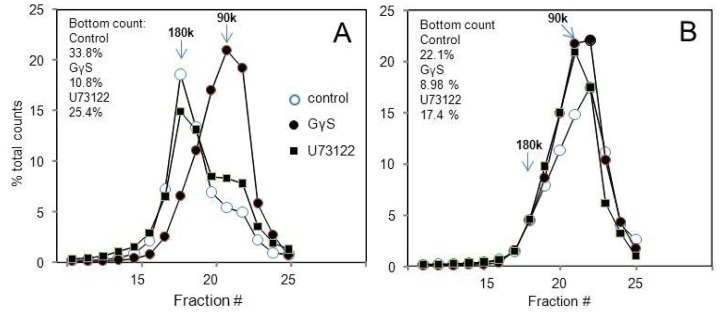
Y2 receptor aggregates in particulates from rabbit tissues yield mainly Gi-Y2 monomer in response to GγS but largely the heteropentamer upon treatment by U73122. Particulates from rabbit kidney (**A**) and anterior hypothalamus (**B**) were incubated with 20 μM GγS or 10 μM U73122 for 15 min at 24 °C, sedimented, resuspended, labeled with 50 pM [^125^*I*]hPYY(3–36) for 15 min at 24 °C, resedimented, lysed at 10 mM each digitonin and cholate and sedimented through a 10%–30% linear sucrose gradient for 6 h at 218,000× *g* and 5 °C, followed by gradient fractionation and counting.

**Figure 6.**
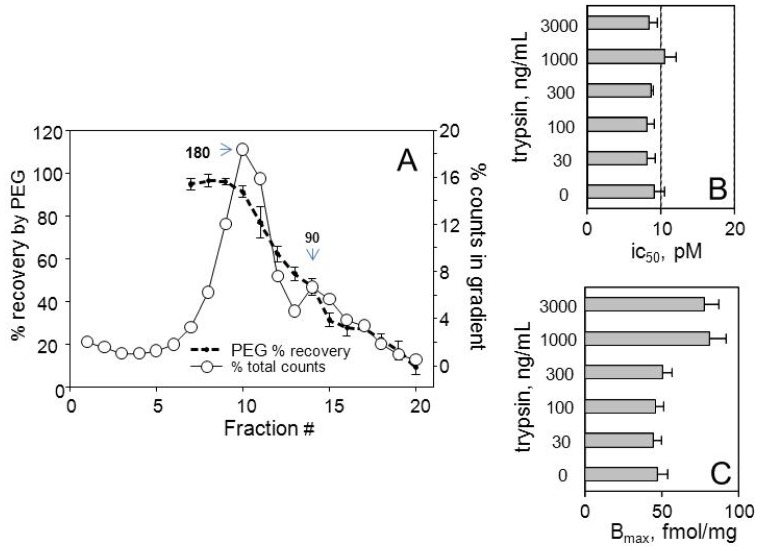
Stability and affinity of agonist binding differs greatly for heteropentamer and monomer forms of the rabbit kidney Y2 receptor. (**A**) Y2 agonist attached to 180 kDa heteropentamer is recovered completely by 10% polyethyleneglycol (PEG) at 0.3 M K^+^ (The data are averages of six gradients, ±1 S.E.). The ic50 for PYY(3–36) binding for the entire receptor complement varies between 0.2 and 0.5 nM, depending on proportions of agonist bound to 180 and 90 kDa complex, and detached from the monomer-Gi α during sedimentation. Note that there is no free intact or partially degraded [^125^*I*]PYY(3–36) in particulates before lysis by digitonin and cholate, and degradation of the receptor-bound tracer is very low; (**B**) Y2 agonist bound to the 90 kDa monomer-Gi α is detached by 8 mM cholate at 0–5 °C (or by 0.3 M KCl) and the affinity of PYY(3–36) binding to the >90% dimeric residue is unaffected by trypsin at up to 3 μg/mL and has ic50 for PYY(3–36) close to 10 pM; (**C**) The *B*_max_ for the dimeric Y2 in the cholate residue increases significantly above 0.3 μg trypsin, indicating a proteolytic disaggregation of the agonist-inaccessible receptor.

More than 30% of the Y2 binding in rabbit kidney postmitochondrial particulates, and more than 20% in the CHO-Y2 postnuclear particulates sediments to the bottom of 5%–20% sucrose gradients over 18 h at 218,000× *g*_max_, corresponding to weight in excess of 1000 kDa. A large part of this material sediments at 30,000× *g*_max_ over 10 min, and could be considered as aggregated. Trypsinization experiments (e.g., Figure 6) show that most of this material could be released as heteropentamers. A considerable part is also releasable by treatment with 20 μM GγS, largely in the form of activated monomers (Figure 5). About a third is released with phospholipase C-blocking alkylating aminosteroid U73122 (10 μM), and this material also sediments in the 90 kDa zone. This preliminary experiment points to potential of such procedures in quantitation of effector-associated receptors at the level of dimers and monomers. There also is a promise of gaining more insight on the size and significance of receptor oligomers incorporating more than two receptor monomers.

The stability and affinity of agonist association with CHO-Y2 receptors could also be analyzed at the level of dimers and monomers by extraction and precipitation procedures coupled to competition binding assays (Figure 6). With rabbit kidney particulates the weak anionic surfactant sodium cholate removes activated monomers, and the heteropentamers show considerable stability to trypsin (graphs B,C), and above 0.3 μg/mL of the enzyme also are enriched in material of the same affinity (obviously deriving from the aggregates examined in Figure 5). The 180 kDa labeled material is not sensitive to 0.3 M K^+^ (as KCl or KI) and precipitates by more than 80% at 10% PEG. The 90 kDa zone agonist binding is sensitive to 0.3 M K^+^, and completely dissociates at 10 mM of zwitterionic surfactant CHAPS, to which the 180 kDa binding is much less sensitive [18].

The above findings show that the entire cycle of signal transduction can be followed, at least for the Y receptors, without use of proteinaceous tags. The G-protein α subunit labeling by GγS is stable at 4 °C, and the βγ subunits and effectors such as cyclases and phospholipases should be assayable using the corresponding antibodies. Also, very similar results are obtained for CHO-Y2 clonal expression and the native rabbit kidney Y2 receptor [24,53], supporting the CHO expression as a physiological model.

### 2.4. Aggregative Clustering Aided by Arrestins Should Help GPCR Cycling

Receptor oligomerization is not linked to aggregative clustering and patching that precede the internalization of most membrane receptors. This aggregation significantly depends on molecules of the arrestin type. Arrestins have a plethora of homoionic clusters (here abbreviated as PCNs, for their abundance in Polynucleotide-associating proteins, protein Carriers (such as importins), and Nuclear localization signals), occupying more than 10% of sequence length, which in most cases quite exceeds the corresponding clusters in Gα subunits (Table 1). The homoacidic segments of arrestins would help in the displacement of G-proteins linked to basic intracellular segments of GPCRs (e.g., those at the *C*-terminus of intracellular loop 3 (ic3) [54]. The numerous homobasic motifs of arrestins in turn should help aggregation of G-protein-free receptors, and transfer of the aggregates into endosomal rafts. In the absence of dephosphorylation the aggregates would translocate into the slow perinuclear path of recycling/disposal [55,56]. However, with raft-level dephosphorylation the receptors can have a fast reassociation with G-proteins and reinsertion into the plasma membrane. It is currently unclear if there could be a significant dissociation and reassociation of Gαβγ and receptor monomers or dimers at the level of the plasma membrane bilayer. As seen in Table 1, Gq α subunit equals β-arrestins in the number of aPCNs, and in this connection there could be a lower arrestin internalization activity with some of Gq-coupling receptors [57]. How the dephosphorylation may affect the oligomeric status of GPCRs was thus far not studied directly.

### 2.5. Hydrophobic Transmembrane Motifs and GPCR Dimerization

**Figure 7.**
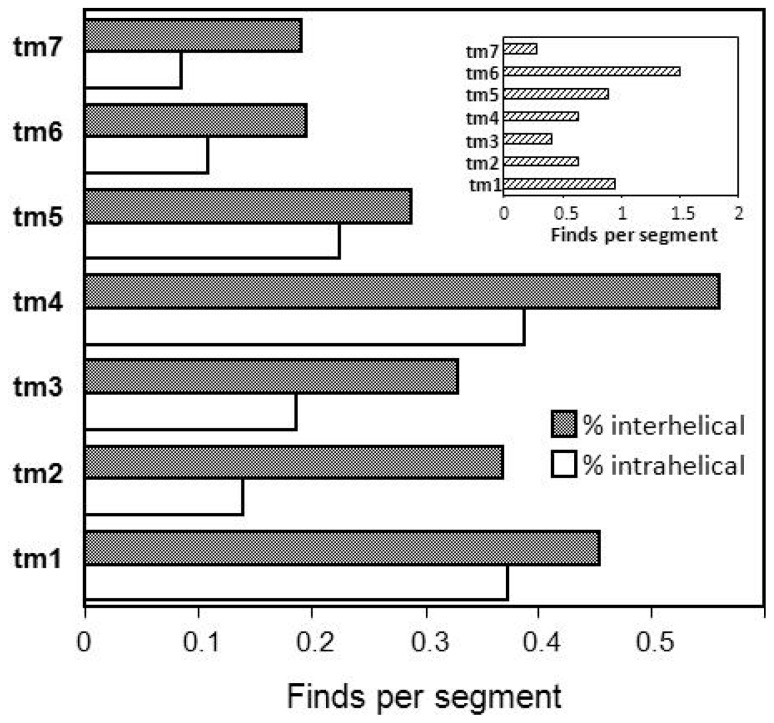
The intrahelical and interhelical hydrogen bonding preferences for transmembrane helices of 209 human A-GPCRs. The triplets with intrahelical and interhelical H-bonding propensity [56] are, respectively, AGF, AGG, GLL, GFF, AGL, ALL, ALS, AGV, AAL, GLV, and AAA, AAG, AAI, AAL, AAM, AFS, AGF, AGG, AGL, AGV, AHS, AIM, AIP, ALF, ALS, GGF, GGL, GGV, GHT, GLF, GLL, HHV, IIL, LLS, LSV. The *inset* shows frequencies of 24 bulky aliphatic hydrophobic triplets corresponding to permutations of Ile, Leu, Met and Val residues.

A general scheme of transmembrane triplet H bonding preferences [58] could be useful in prediction of intra- and interhelical matches in GPCRs (Figure 7). The tm4 and tm1, which are frequently identified as oligomer formers, stand out in the abundance of both intra- and interhelical strongly H-bonding triplets. Tm6, tm1 and tm5 lead in the bulky aliphatic hydrophobic triplets (the inset of Figure 7). These abundances generally correspond with reports about use of the tm helices in dimerization of GPCRs.

There could be a spatial dimerization preference for use of the tm1, tm4 and tm5 helices, which are somewhat apart from other domains in the rhodopsin transmembrane bundle [1]. The tm1 leads in hydrophobicity and the tm6 in bulky neutral residues (Figure 7). The above parameters support assemblies like tm1:tm4 for the α1B-adrenergic receptor [59], tm4:tm4 for the D2 dopamine receptor and bovine rhodopsin [3], tm4:tm5 for bovine rhodopsin [4] and the A3 adenosine receptor [60]. A helix bundling based on the tm6 could be present for the cholecystokinin-A (CCK-A) receptor [61].

Long hydrophobic stretches are known to support transmembrane dimerization [62,63], especially if entailing GXXXG motifs. The GXXXG motifs are known to be important in dimerization of glycophorins (major stabilizers of erythrocyte membranes) and of other membrane proteins [64,65]. This motif is present in 66 of 1477 transmembrane domains in 211 examined human A-GPCRs (4.5%), and in more than 5% sequences of tm4 and tm6 helices (Table 2). The adrenergic α1A and α1B receptors have GXXXG motifs in tm1, tm4 and tm6 domains, FFA1 receptor in tm3, tm4 and tm7 segments, and the orphan masE in tm1 and tm6 helices. The α1A and α1B adrenergic receptors are known to form stable homo- and heterodimers [66], but FFA1 and masE receptor dimerization does not seem to have been studied thus far.

The extended LXXXGXXXG motif, a stronger indicator of transmembrane dimerization, is however found in only seven transmembrane domains of A-GPCRs. The respective receptors include homo- and hetero-oligomerizing CC4 and Duffy [67] chemokine receptors, and FFA1, galanin-2, bile acid-1, neuromedin-U2, P2Y11 purinergic and sphingosine-1-phosphate receptor Edg-6, for any of which there are no direct oligomerization reports.

All transmembrane domains of 211 human A-GPCRs and whole sequences of human glycophorins A and C were examined for presence of GXXXG motifs, and of ILMV and FWY as XXX residues in the motifs. As seen in Table 2, the tm6 and tm4 domains have the most candidate GXXXG motifs, and tm7 and tm1 the fewest. The large aliphatic hydrophobic XXX residues are clearly in excess of other except in tm3, which has a low ILMV and a strong aromatic sidechain presence in 10 GXXXG motifs.

### 2.6. Association of Intracellular Basic Clusters with Transducers Could Support Dimerization of GPCRs

Intracellular domains of heptahelical GPCRs consist of three loops, one juxtamembrane helix (helix 8, H8) and a *C*-terminal largely unstructured region [1]. As seen in Table 3, all of these intracellular parts have a quite basic character. This would help prevent intramolecular or intermolecular contacts and interactions between these domains. This in particular applies to the first two loops, which also are quite short and homogenous in size. The third loop is highly differentiated in size and ionic motifs [68], and has undergone a very extensive expansion in many subgroups of GPCRs, and especially in aminergic receptors [69]. The juxtamembrane intracellular helix H8 following the seventh transmembrane domain is essentially a homobasic tract (Table 3). Ionic interactions are generally important for association of Gαβγ with effectors [70].

As seen in Table 3, all subunits of heterotrimeric G-proteins (which are the most frequently detected partners of dimeric GPCRs) have similar sequence fractions of acidic and basic sidechains and homoacidic segments occupy consistently larger parts of sequence than the homobasic. The (infrequent) basic clusters are however more represented than acidic. These features point to a large potential for association with homobasic tracts in partner GPCRs (and possibly also with homoacidic tracts of effectors). All intracellular (ic) domains of A-GPCRs (the rhodopsin family non-visual GPCRs) are quite rich in basic, and low in acidic residues. The short loops 1 and 2 are quite close in basicity to the strongly basic human 60S ribosomal proteins (Table 3). Similar applies to homoionic segments and PCN clusters, and there are no acidic clusters in ic1 and ic2 loops. The *C*-terminal section of the ic3 loop (which is frequently identified as partner of Gα subunits [54,71–73]) is quite more basic than the entire ic3 loop, but also contains an acidic residue that could function as a switch. (Note that most A-GPCRs have at least 13 aa well aligned in the ic3 loop, and these are positioned at the *C*-terminus in longer ic3 sequences, and represent a helix coextensive with tm6 [69]). Helix 8 (H8) in the ic4 domain is considerably above the entire domain in basicity, homoionic segregation and content of basic clusters (Table 3). This area could extensively interact with G-proteins, perhaps as a factor in dimerization [6,74] as is also indicated by several modeling studies employing artificial bilayers [5,42,75]. The H8 domains lacking CC pair at the *C*-terminus are more frequent in A-GPCRs, and receptors from the olfactory, Taste-2, secretin (family B) and ion-sensing (family C) GPCRs do not have cysteine in the juxtamembrane intracellular 20-residue segment abutting the tm7.

### 2.7. The Transductionally Stable C-GPCR Dimers Point to Possibly Similar Design in Other GPCR Groups

The cation (calcium)-sensing, glutamate and γ-aminobutyrate type B (GABAB) family C receptors and Taste-1 receptors all have large *N*-terminal exocellular domains and an obligatory covalent dimerization via cysteine bridges, which mainly serves to help shaping and maintenance of the elaborate ligand binding nests (“Venus fly-trap” sites; see e.g., [76]). As different from A-GPCRs, the transduction to Gi or Gq α subunits does not result in a fast dimer dissociation or internalization. However, in response to agonist nesting there are transmembrane movements [77] leading to effector activation. The accessory homer proteins have strong aPCNs and also bPCNs, and could act similar to arrestins in separation of Gα from the receptor intracellular domains. The C-GPCR interaction with homers via the ic4 could reduce mobility of transmembrane domains and help maintenance of transmembrane dimers. Dimerization of C-GPCRs thus has a stable principal component which aids the maintenance of the ligand binding site, and an auxiliary transmembrane component which may function in a pulsatile manner to help resetting of the Gα nucleotide site.

The above type of dimer response to agonist could be to some degree present with all dimerizing GPCRs. Dephosphorylation of GTP that powers-up the transduction may not necessarily result in Gα detachment from the receptor, especially not if ligand attached to the receptor retains sufficient affinity after triggering of the nucleotide site. Such a retention would be expected for large peptide agonists with strong basic motifs, such as apelin (Q9ULZ1), ghrelin (Q9UBU3) and NPY (P01303). This may enhance the transductional versatility of dimers across the GPCR families. Dimers of glycoprotein hormone A-GPCRs with large *N*-terminal extracellular domains deserve special notice. As with C-GPCRs, ec1 Cys residues are important in receptor activity of follicle-stimulating hormone (FSH; [78]), luteinizing hormone (LH; [79]) and thyrotropin (TSH; [80]). The receptors achieve active conformation via stepwise and asymmetric occupancy of binding sites on the two protomers by >200 aa agonist proteins, with a negative cooperativity [81] and protomer-specific messaging to Gq α and to Gβγ [82]. The size of agonist peptides as well as of the binding sites could be expected to help a prolonged receptor occupancy and might support extended transduction, probably terminated by internalization of the receptor-ligand complex. Receptors that have low internalization rates could also fit in this category. This could include NPY Y2 [83], angiotensin AT2 [84], bradykinin Bk1 [85], somatostatin sstr2 [86] and opioid δ [87] subtypes, which all counter-match immediate-response, fast-internalizing parallel subtypes.

## 3. Experimental Section

### 3.1. Materials

Human Y receptor cDNAs packaged in Invitrogen pcDNA 3.1+ vector were donated by the University of Missouri at Rolla (MO, USA). The receptors were stably expressed in CHO-K1 cells (from the American Type Culture Collection, Baltimore, MD, USA). Human/rat neuropeptide Y (NPY), porcine/rat peptide YY (pPYY), human peptide YY(3–36) (PYY(3–36)) and human pancreatic polypeptide (hPP) were obtained from the American Peptide Company (Sunnyvale, CA, USA), or from Bachem (King of Prussia, PA, USA). The Y2 antagonist BIIE0246 was purchased from Tocris (Ellisville, MD, USA). Rabbit antibodies against human Gi and Gq α subunits were from Upstate (Lake Placid, NY, USA). Monoiodinated HPLC-purified [^125^*I*]human PYY(3–36) (hPYY(3–36)) was from Phoenix Pharmaceuticals (Shadyvale, CA, USA), while [^125^*I*] human neuropeptide Y (NPY), porcine/rat peptide YY (pPYY) and human pancreatic polypeptide (hPP) were from PerkinElmer (Cambridge, MA, USA). Guanosine 5′-*O*-(γ-thiotriphosphate) (GγS) labeled by ^35^*S* was from PerkinElmer. Digitonin (high purity) was from Calbiochem (La Jolla, CA, USA). All other chemicals were from Sigma (St. Louis, MO, USA). Spin columns were from Pierce (Rockford, IL, USA).

### 3.2. Receptor and Nucleotide Site Labeling

The receptors were labeled for 30 min at 25 °C in respective particulates (50 μg protein/assay), employing not more than 50 pM [^125^*I*]-labeled agonist peptides (as stated in individual experiments). Higher molar inputs of agonist peptides would produce significant Gα activation within short exposures (e.g., [24,53]). The basal assay buffer contained 0.2% proteinase-free bovine serum albumin, 20 mM hepes.NaOH (pH 7.4), 1 mM diisopropylfluorophosphate, 0.04% bacitracin, and 10 μg/mL each of proteinase inhibitors aprotinin, bestatin, chymostatin, leupeptin and pepstatin. Depending on experiment design, this was supplemented by 3 mM CaCl_2_ and 1 mM MgCl_2_, (buffer A) or by 1 mM Na EDTA, pH 7.4 (buffer B). The nucleotide site labeling at 200 pM [^35^*S*]GγS was for 30 min at 28 °C after pre-activation for 30 min at 28 °C at 3 μM GDP in the basal buffer with 50 μM EDTA, 100 mM NaCl and 4 mM MgCl_2_, and without CaCl_2_ (buffer C). All subsequent operations were carried out at 0–5 °C. The assays were terminated by sedimentation for 10 min at 30,000× *g*_max_, followed by surface wash of the pellets.

Precipitation with polyethyleneglycol-8000 (PEG; 10% final) was done with aliquots of gradient fractions at 300 mM KCl or KI and 50 μg/mL bovine γ-globulin, for 10 min at 0–4 °C, followed by sedimentation for 10 min at 1200× *g* (5 °C). The pellets were then counted in a scintillation counter.

### 3.3. Characterization of Receptors in Density Gradients

Rabbit kidney postmitochondrial particulates, rabbit piriform cortex and hypothalamic crude synaptosomes and CHO cell postnuclear particulates were prepared as described previously [24,53]. After solubilization of particulates at 10 mM each of digitonin and sodium cholate in buffer A or B, the lysates were spun for 10 min at 1000× *g* to remove debris, and the supernatants sedimented for periods indicated in figure captions at 218,000× *g*_max_ through linear 5%–20% sucrose gradients (10 mL, made in buffer A). The gradients were divided by bottom dripping in 23 fractions of 0.45 mL. The sedimentation positions were calibrated with [^125^*I*]-labeled bovine γ-globulin (158 kDa), human iron-saturated transferrin (75 kDa) and ovalbumin (44 kDa), and with colorized myosin (211 kDa), producing a linear relation of migration to molecular weight (*R*^2^ > 0.99).

### 3.4. Immunodetection of G-Protein α-Subunits Coupled to Receptors

For immunodetection of G-protein α subunits and associated receptors, aliquots of [^125^*I*] agonist-labeled fractions from gradients (100 μL) were incubated with antibodies to Gi3 or Gq α subunits (at 1:250 final dilution) for 12 h at 5 °C. Protein A/protein G agarose was then added (50 μL/250 μL final volume), the mixtures were rotated for 6 h at 4 °C, loaded onto spin columns and spin-washed with 2 × 1 mL of the cold buffer C prior to counting of the gels in a scintillation counter.

## 4. Conclusions

The Y receptor heteropentamers poorly respond to guanosine triphosphates in the absence of agonists, but can be converted to monomers by high inputs of either receptor or nucleotide site agonists; this may apply to most GPCRs that respond to peptidic agonists. Using the gradient centrifugation or gel chromatography, metabolic processing in the presence of Mg^2+^ could dissect physical stages in dimer utilization, including modes of association with Gα and βγ subunits, effector enzymes and ion transporters and exchangers. The arrestin-assisted dimer disassembly and reassembly could use the perinuclear or the trans-Golgi endosomal route. The cell and tissue levels of GPCR non-covalent transmembrane heteropentamers and larger oligomers could be inversely related to those of heterotrimeric G-proteins.

Family C GPCRs and the slowly internalizing peptide A-GPCRs could stabilize dimers via both non-transmembrane interactions and transmembrane domain links to achieve repeated use of the same dimer.

With Y receptors as models, heteropentamers resemble a storage form of receptors, equipped with transducers and ready to handle large inputs of agonist peptides. This type of organization could be expected for all GPCRs that respond to peptidic agonists secreted in discharges.

## Figures and Tables

**Table 1.** Comparison of homoionic clusters in G-protein α subunits and β-arrestins.

Molecule	Number of aa	aPCN count	% aPCN	bPCN count	% bPCN
Go	354	2	1.98	3	3.39
Gi1	354	3	3.11	4	4.24
Gi2	355	3	3.10	3	3.10
Gi3	354	3	3.11	4	4.24
Gt1	350	2	2.29	5	6.29
Gt2	354	2	2.26	4	5.09
Gt3	354	2	2.26	3	3.39
Gq	353	4	4.82	3	3.12
Gs	394	2	1.52	2	2.03
Golf	381	1	0.787	4	4.2
β-arrestin-1	418	4	4.07	6	6.70
β-arrestin-2	409	4	4.16	6	6.85

% aPCN, % bPCN Segments with ≥3% and ≥50% acidic or basic residues as % sequence amino acids (aa).

**Table 2.** GXXXG motifs in transmembrane domains of rhodopsin family human GPCRs and glycophorins.

Domain	% Sequences with GXXXG	% ILMV in XXX	% FWY in XXX
tm1	2.84	40	0
tm2	0.948	30	0
tm3	4.74	16.4	25.5
tm4	7.11	38.7	9.33
tm5	3.79	35	7.50
tm6	10.43	46.4	6.36
tm7	0.474	40	0
Glycophorins	100	50	50

**Table 3.** A comparison of ionic constituents in human G-proteins, intracellular domains of A-GPCRs and 60S ribosomal proteins.

Group	Mean # aa	DE %	HKR %	Homo-acidic %	Homo-basic %	aPCN %	bPCN %
**G-protein subunits**							

Gα [17]	362	15	15.5	27.1	27.9	2.30	3.88
Gβ [5]	351	11.6	12.0	28.3	20.0	0.15	1.70
Gγ [11]	71	13.9	15.5	25.3	20.8	0.54	0

**A-GPCRs (rhodopsin family)**							

ic1 [211]	11.2	1.68	25.9	0.30	39.6	0	17.1
ic2 [211]	14.8	1.69	26.0	0.50	58.1	0	10.8
ic3 [211]	42.9	5.56	27.5	5.22	62.0	0.64	13.2
ic3 *C*-terminal 13 aa [211]	13.0	5.90	35.5	1.87	62.1	0.51	17.3
ic3 less *C*-terminal 13 aa [207]	31.4	4.88	21.4	5.32	42.5	0.52	6.66
ic4 [211]	56.5	9.23	18.4	12.3	44.1	1.26	4.73
H8 [210]	14.0	6.22	24.5	1.13	41.3	0.10	6.49
ic4 past H8 [208]	39.5	9.72	16.1	14.8	30.9	1.51	3.91

**Ribosomal proteins**							

60S ribosomal proteins [49]	168	7.27	28.2	8.00	66.2	0.85	18.2

All values are means of the respective parameters. All percentages in headers refer to lengths of the respective (segment or whole molecule) sequences. Numbers of sequences examined are shown in brackets after group labels. Homoacidic and homobasic percentages represent fractions of sequence delimited by acidic or basic residues, and containing two or more such residues. Terms “aPCN” and “bPCN” are defined in the footnote to Table 1.
